# On the Parameterized Complexity of Compact Set Packing

**DOI:** 10.1007/s00453-024-01269-6

**Published:** 2024-09-13

**Authors:** Ameet Gadekar

**Affiliations:** Department of Computer Science, Bar-Ilan University, Ramat Gan, Israel

**Keywords:** Parameterized complexity, Set packing

## Abstract

The Set Packing problem is, given a collection of sets $\mathcal {S}$ over a ground set *U*, to find a maximum collection of sets that are pairwise disjoint. The problem is among the most fundamental NP-hard optimization problems that have been studied extensively in various computational regimes. The focus of this work is on parameterized complexity, Parameterized Set Packing (PSP): Given parameter $r \in {\mathbb N}$, is there a collection $\mathcal {S}' \subseteq \mathcal {S}: |\mathcal {S}'| = r$ such that the sets in $\mathcal {S}'$ are pairwise disjoint? Unfortunately, the problem is not fixed parameter tractable unless $\textsf {W[1]} = \textsf {FPT}$, and, in fact, an “enumerative” running time of $|\mathcal {S}|^{\Omega (r)}$ is required unless the exponential time hypothesis (ETH) fails. This paper is a quest for tractable instances of Set Packing from parameterized complexity perspectives. We say that the input $({U},\mathcal {S})$ is “compact” if $|{U}| = f(r)\cdot \textsf {poly} ( \log |\mathcal {S}|)$, for some $f(r) \ge r$. In the Compact PSP problem, we are given a compact instance of PSP. In this direction, we present a “dichotomy” result of PSP: When $|{U}| = f(r)\cdot o(\log |\mathcal {S}|)$, PSP is in FPT, while for $|{U}| = r\cdot \Theta (\log (|\mathcal {S}|))$, the problem is W[1]-hard; moreover, assuming ETH, Compact PSP does not admit $|\mathcal {S}|^{o(r/\log r)}$ time algorithm even when $|{U}| = r\cdot \Theta (\log (|\mathcal {S}|))$. Although certain results in the literature imply hardness of compact versions of related problems such as Set $r$-Covering and Exact $r$-Covering, these constructions fail to extend to Compact PSP. A novel contribution of our work is the identification and construction of a gadget, which we call Compatible Intersecting Set System pair, that is crucial in obtaining the hardness result for Compact PSP. Finally, our framework can be extended to obtain improved running time lower bounds for Compact $r$-VectorSum.

## Introduction

Given a graph $G = (V, E)$, the problem of finding a maximum-size subset of disjoint edges (matching) is tractable, but its generalization to hypergraphs, even when the edge length is 3, is NP-hard. This general problem is known as the Hypergraph Matching problem. The hyper-graph $H=(W,F)$ can be equivalently viewed as a set system $({U},\mathcal {S})$, where the universe (or the ground set) *U* corresponds to the vertex set *W* and $\mathcal {S}$ corresponds to the collection of hyperedges *F*. Then finding a maximum matching in *H* is equivalent to finding maximum number of pairwise disjoint sets (packing) in $\mathcal {S}$. Hence the Hypergraph Matching problem is also known as the Set Packing problem, which is a fundamental problem in combinatorial optimization with numerous applications. While this problem captures many classical combinatorial problems such as maximum independent set (or maximum clique), *k*-dimensional matching and also, some graph packing problems [1, 2], this generalization also makes it intractable in several regimes. One computational regime in which Set Packing has been explored extensively is approximation algorithms. Since Set Packing generalizes the maximum independent set problem [3], it inherits the inapproximability of the latter problem [4]. This immediately implies that the trivial approximation of picking simply one set in the packing is roughly the best to hope for. Furthermore, approximations in terms of |*U*| are also not hopeful since the result also implies inapproximability bound of $|{U}|^{1/2 -\epsilon }$, which is matched by [5]. To combat these intractabilities, various restrictions of Set Packing have been studied. Particularly, a restriction where the size of the sets in $\mathcal {S}$ is bounded by some integer *k*, which is known as $k$-Set Packing, is also a well-studied problem. However, $k$-Set Packing captures the independent set problem in bounded degree graphs, which again is a notoriously hard problem to approximate beyond the “trivial” bound [6, 7]. While [8] improves the lower bound for $k$-Set Packing to $\Omega (k/\ln k)$, the best known approximation is $(k+1+\epsilon )/3$ [9, 10], yielding a logarithmic gap between the bounds. Besides approximation algorithms, Set Packing has also been studied from the parameterized complexity perspectives (with the standard parameter on the size of an optimal packing solution). In this problem, known as the Parameterized Set Packing (PSP) problem, we are given a set system $({U},\mathcal {S})$ and a parameter $r \in {\mathbb {N}}$, and the task is to decide if there exists a packing of size *r*. Unfortunately, even PSP remains intractable and is, actually, W[1]-complete [11]. In fact, *Exponential Time Hypothesis* (ETH) implies that the trivial *enumerative algorithm* running in $O^{*}(|\mathcal {S}|^r)$ time to find an *r*-packing is asymptotically our best hope [12]. The algorithmic outlook for PSP worsens further due to [13], which rules out *o*(*r*)-FPT-approximation algorithm assuming the *Gap Exponential Time Hypothesis* (Gap-ETH).

Assuming a weaker hypothesis of $\textsf {FPT} \ne \textsf {W[1]}$, very recently [14] showed that there is no FPT algorithm for PSP problem that finds a packing of size $r/r^{1/H(r)}$, for any increasing function $H(\cdot )$, when given a promise that there is an *r*-packing in the instance. Thus, the flurry of these negative results make it likely that Set Packing problem is intractable in all computational regimes.

In this paper, we consider PSP on *compact* instances. We say that an instance $$({U}, \mathcal {S}, r)$$ of $${PSP} $$ is compact if $$|{U}| = f(r) \cdot \textsf {poly} (\log |\mathcal {S}|)$$, for some function $$f(r) \ge r$$, that is, the universe is relatively small compared to the number of sets^1^. Besides the algorithmic motivation, compact instances have recently been used as an “intermediate step” to prove FPT inapproximability results of the (non-compact) classical problems (see, e.g., [16, 17] where the compact instances were used in proving FPT-inapproximability of the *k*-EvenSet and Dominating Set). We hope that studying Compact PSP would lead to some ideas that would be useful in proving tight FPT inapproximability of PSP (that is, to weaken the Gap-ETH assumption used in [13]).

### Our Results

Our main result is the following dichotomy of Parameterized Set Packing.

#### Theorem 1

(Dichotomy) The following dichotomy holds for PSP.

$$\circ $$
If $|{U}| = f(r)\cdot o(\log |\mathcal {S}|)$, for any *f*, then PSP is in FPT.
$$\circ $$
PSP remains W[1]-hard even when $|{U}| = r \cdot \Theta (\log |\mathcal {S}|)$.

The algorithmic result follows from well-known dynamic programming based algorithms [12, 18] that run in time $O^*(2^{|{U}|})$, and observing that this running time is fixed parameter tractable [15] when $|{U}| = f(r)o(\log |\mathcal {S}|)$.

The main contribution of our work is the W[1]-hardness of PSP even when $|{U}| = r \cdot \Theta (\log |\mathcal {S}|)$. Towards this, we show an FPT-reduction from $k$-Clique to Compact PSP. The hardness result follows since $k$-Clique is W[1]-hard. We remark that the classical reduction given of [11] has |*U*| linear in $|\mathcal {S}|$. In fact, using an intricate reduction from Subgraph Isomorphism (SGI), our hardness result can be strengthened assuming Exponential Time Hypothesis (ETH) [12] to obtain the following result.

#### Theorem 2

PSP requires time $|\mathcal {S}|^{\Omega \left( r/\log r\right) }$ even when $|{U}| = r \cdot \Theta (\log |\mathcal {S}|)$, unless ETH fails.

The result of Theorem 2 follows from the ETH-hardness result of SGI due to [19], and from the fact that the hardness reduction of Theorem 2 is parameter preserving up to a multiplicative constant. Note that since PSP can be trivially solved by enumeration in time $O^*\left( |\mathcal {S}|^r\right)$, the above result says that, even for the compact instances this is essentially our best hope, up to a $\log$ factor in the exponent. An interesting consequence of the dichotomy theorem coupled with Theorem 2 is the fact that, for the instances whose universe size is asymptotically smaller than that of the compact instances, not only we beat the enumerative algorithm, but we actually obtain an FPT algorithm. We would like to remark that the universe size in Theorem 2 is tight (up-to $\log r$ factor) since having $|{U}| = o(r/\log r) \cdot \Theta (\log |\mathcal {S}|)$ would already yield a $|\mathcal {S}|^{o(r/\log r)}$ time algorithm. Further, note that to show W[1]-hardness, it is sufficient to have $|{U}| = f(r) \cdot \Theta (\log |\mathcal {S}|)$, for some *f*, since we can add $f(r)-r$ new sets each with a unique dummy element and inflate the parameter to *f*(*r*). However, this is not true for ETH based running time lower bounds as such inflation fail to transfer the lower bounds asymptotically.

We would like to point out that, after our article was made public, Huairui Chu posted an article [20] improving the lower bound of Theorem 2 to rule out $|\mathcal {S}|^{\Omega (r)}$ time for Compact PSP.

Finally, we extend our construction framework (Theorem 3) to improve the running time lower bound (matching the trivial upper bound up to a $\log$ factor in the exponent) for the compact version of $r$-VectorSum: Given a collection $\mathcal {C}$ of *N* vectors in ${\mathbb {F}}^d_2$, and a target vector $\vec {b} \in {\mathbb {F}}^d_2$, $r$-VectorSum asks if there are *r* vectors in $\mathcal {C}$ that sum to $\vec {b}$. Compact $r$-VectorSum is defined when $d = f(r) \cdot \textsf {poly} (\log N)$, for some $f(r)\ge r$.

#### Theorem 3

$r$-VectorSum requires time $N^{\Omega (r/\log r)}$, even when $d=r \cdot \Theta (\log N)$, unless ETH fails.

The present bound of [16] rules out $N^{o(\sqrt{r})}$ time under ETH.

The proof of this theorem is present in Sect. 5.

### Our contributions and comparison to existing works

In this section, we compare our contribution with existing works to highlight its significance. To our best knowledge, the compact version of combinatorial problems considered in this paper has not previously been formalized and investigated. However, several existing reductions already imply the hardness of compact version of some of the combinatorial problems. Here we review and compare the related results.

**Our Contribution.** As far as we know, there are no results showing W[1]-hardness of Compact PSP, and hence the corresponding dichotomy (Theorem 1). The key contribution of this paper is to show the hardness result for Compact PSP.

On the way, we also show an ETH-based almost tight running time lower bound for Compact PSP, with tight (up-to $\log r$ factor) universe size $|{U}|= r\cdot \Theta (\log |\mathcal {S}|)$. Interestingly, we show both of these results with a single FPT reduction. In addition, we extend our framework to improve the running time lower bounds for Compact $r$-VectorSum.

Next we survey some known hardness results for Set $r$-Covering in the compact regime and argue their limitations in extending them to PSP. In particular, [21, Lemma 25] shows a reduction from SGI, where we are given two graphs $G=(V_G,E_G)$ and $H=(V_H,E_H)$, and an integer *k* such that $|E_H|=k$ and asked to find an isomorphic (not necessary induced) copy of *H* in *G*, to a variant of Set $r$-Covering called Compact Exact $r$-Covering , where we want to find an *r*-packing that is also a covering (in fact they show hardness for Compact Set $r$-Covering . But a closer inspection of their construction shows that the intended set cover is also a packing). The high level idea of the construction is similar to ours: first assign each vertex of *G* a logarithm length binary pattern vector. Then, create two kinds of sets: *V*-sets that capture the vertices of the subgraph $G'$ of *G* that is isomorphic to *H* and *E*-sets that capture the edges of $G'$. The idea is to use the pattern vectors to create these sets so that there is an isomorphic copy of *H* in *G* if and only if there are $|V_H|$ many *V*-sets and $|E_H|$ many *E*-sets covering the universe exactly once. However, if we consider the Soundness (No case) proof of this reduction, then it crucially relies on the fact that no candidate solution can cover the entire universe exactly once. In fact, it is quite easy to find *r* sets that are mutually disjoint but do not form a cover.

Therefore, it fails to yield hardness for Compact PSP. The heart of our construction lies in ensuring that in the No case, any *r* sets intersect. To this end, we construct a combinatorial gadget called Compatible Intersecting Set System (ISS) pair. This gadget is a pair of set systems $({{\mathbb {A}}},{{\mathbb {B}}})$ over a universe *U* that guarantees two properties: First, every pair of sets within each set system intersects, and second, for any set $a \in {{\mathbb {A}}}$, there exists $b\in {{\mathbb {B}}}$ such that *a* intersects every set in ${{\mathbb {B}}}$ except *b*. Further, we present a simple greedy algorithm that finds such compatible ISS pair $({{\mathbb {A}}},{{\mathbb {B}}})$ over a universe of size *N*, each having roughly $2^{\Omega (N)}$ sets. Note that this gadget, which we use to build our compact hard instance, also has a “compact” universe.

While, on the other hand, [22] shows Compact Set $r$-Covering is W[1]-hard using a reduction from $k$-Clique to Set $r$-Covering with $r=\Theta (k^2)$ and $|{U}| = r^{3/2} \cdot \Theta (\log |\mathcal {S}|)$, but does not yield a tight ETH- based running time lower bound.

In contrast, [23] shows such tight ETH lower bound for Compact Set $r$-Covering : requiring time $|\mathcal {S}|^{\Omega \left( r\right) }$, which can be easily modified to obtain a similar running time lower bound for Compact Exact $r$-Covering (by reducing from $1$-in- $3$-SAT, instead from $3$-SAT).

### Open Problems

An interesting direction is FPT approximating Compact PSP: Given a promise that there is an *r*-packing, is it possible to find a packing of size $\omega (1)$ in FPT time? Note that for the general PSP problem, there is no *o*(*r*) FPT-approximation, assuming Gap-ETH. However, recent results [14, 24] use a weaker assumption of $\textsf {W[1]} \ne \textsf {FPT}$ but also obtain weaker FPT-inapproximibility.

It is also interesting to show such hardness of approximation for Compact PSP.

Organization We start with the preliminaries in Sect. 2. The proof of Theorem 1 is presented in Sect. 3. This proof also serves as a warmup for the more intricate proof of Theorem 2, which is presented in Sect. 4. The proof of Theorem 3 is presented in Sect. 5.

## Preliminaries

### Notations

For $q \in {\mathbb {N}}$, denote by [*q*], the set $\{1,\ldots , q\}$. For a finite set [*q*] and $i \in [q]$, we overload ${``}+{"}$ operator and denote by $i+1$ as the (cyclic) successor of *i* in [*q*]. Thus, the successor of *q* is 1 in [*q*].

All the $\log$s are in base 2. For a graph $G=(V,E)$ and a vertex $v \in V$, denote by *N*(*v*), the set of vertices adjacent to *v*. Further, *d*(*v*) denotes the degree of *v*, i.e., $d(v):= |N(v)|$. For a finite universe *U* and $S \subseteq U$, denote by ${\bar{S}}$ as the complement of *S* under *U*, i.e., ${\bar{S}}:= U {\setminus } S$. Similarly, for a family of sets ${\mathcal {S}}=\{S_1,\ldots ,S_M\}$ over *U*, we denote by $\textsf{comp}(\mathcal {S})=\{{\bar{S}}_1,\ldots ,{\bar{S}}_M\}$. Further, for a subset $S \subseteq U$ and a sub-universe $U' \subseteq U$, denote by $S \mid _{U'}$ as the restriction of *S* on sub-universe $U'$, i.e., $S \mid _{U'}:= S \cap U'$. Similarly, for a family of sets ${\mathcal {S}}=\{S_1,\ldots ,S_M\}$ over *U*, denote by $\mathcal {S}\mid _{U'}$ as the restriction of every set of $\mathcal {S}$ on $U'$, i.e., $\mathcal {S}\mid _{U'}:= \{S_1\mid _{U'},\ldots ,S_M\mid _{U'}\}$. For a set system ${{\mathbb {A}}} = (U_A,\mathcal {S}_A)$, we denote the complement set system by $\overline{{{\mathbb {A}}}} = (U_A,\textsf{comp}(\mathcal {S}_A))$. For $S,T \subseteq U$, we say *S* and *T* intersects if $S \cap T \ne \emptyset$. For a variable *n*, $\textsf {poly} (n)$ represents a function $\Theta (n^c)$, for some constant $c \ge 1$.

### Parameterized Complexity

The parameterized complexity theory concerns computational aspects of languages $(L,\kappa )$ over a fixed and finite alphabet $\Sigma$, where $L \subseteq \Sigma ^*$, and $\kappa : \Sigma ^* \rightarrow {\mathbb {N}}$, called the parameter, is a polynomial time computable function.

Thus a parameterized problem is a classical problem together with a parameter $\kappa$. As an example consider the following classical NP-complete problem.
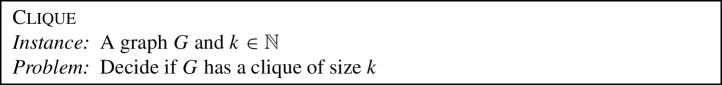


Now consider a parameterized version of Clique defined by $\kappa (G,k):= k$.
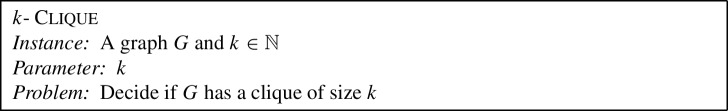


When the parameter $\kappa$ represents the size of solution, then it is called *natural* parameter.

#### Definition 1

(Fixed Parameter Tractable) A parameterized problem $(L,\kappa )$ is called *fixed parameter tractable* if there is an algorithm *A*, a constant *c* and a computable function $f: {\mathbb {N}} \rightarrow {\mathbb {N}}$ such that on all inputs $y=(x,k)$, *A* decides whether *x* is in *L* and runs in time at most $f(k)\cdot |x|^c$.

The complexity class FPT is the set of all fixed parameter tractable problems. In this paper, we consider parameterized problems with natural parameter i.e. $\kappa$ represents the size of solution. Once we define the class FPT, the next natural thing is to define *parameterized reduction* or FPT*-reduction* with the intention that such a reduction from parameterized problem *Q* to another parameterized problem $Q'$ allows converting an FPT algorithm of $Q'$ to an FPT algorithm of *Q*.

#### Definition 2

(FPT-reduction) An FPT*-reduction* from a parameterized problem $Q \subseteq \Sigma ^* \times {\mathbb {N}}$ to a parameterized problem $Q' \subseteq \Sigma ^* \times {\mathbb {N}}$ is an algorithm *R* mapping from $\Sigma ^* \times {\mathbb {N}}$ to $\Sigma ^* \times {\mathbb {N}}$ such that for all $y=(x,k), R(y) \in Q'$ if and only if $y \in Q$, and for some computable function *f* and a constant *c*, *R*(*y*) runs in time $f(k)\cdot |x|^c$ and $R(y)=(x',k')$, where $k'\le g(k)$ for some computable function *g*.

An extensive treatment of the subject can be found in [11, 12, 25].

### Problem definitions

#### Definition 3

(Parameterized Set Packing (PSP)) Given a collection of sets $\mathcal {S}=\{S_1,\dots , S_m\}$ over an universe ${U} = \{e_1,\dots ,e_n\}$ and an integer *r*, the PSP problem asks if there is a collection of sets ${\mathcal {S}}' \subseteq {\mathcal {S}}$ such that $|{\mathcal {S}}'| = r$ and, $S_i \cap S_j = \emptyset$ for every $S_i \ne S_j \in \mathcal {S'}$. An instance of PSP is denoted as $({U},\mathcal {S},r)$.

#### Definition 4

(*f*(*r*)-Compact PSP) We say an instance $(U,\mathcal {S},r)$ of PSP is *f*(*r*)-*compact* for some function $f(r)\ge r$ if $|U| = f(r) \textsf {poly} (\log |\mathcal {S}|)$. *f*(*r*)-Compact PSP is defined when the instance is *f*(*r*)-compact.

#### Definition 5

($k$-Clique) Given a graph $G=(V,E)$ and an integer *k*, the $k$-Clique problem asks if there is a clique of size at least *k* in *G*.

Given two graphs $G=(V_G,E_G)$ and $H=(V_H,E_H)$, a homomorphism from *H* to *G* is a map $\phi : V_H \rightarrow V_G$ such that if $(v_i,v_j) \in E_H$ then $(\phi (v_i), \phi (v_j)) \in E_G$. If $\phi$ is an injective homomorphism then *H* is said to be *isomorphic (not necessary induced)*^2^ to the subgraph of *G* on the vertices in the image of $\phi$. In this paper, for brevity, we will stick to the term isomorphic meaning isomorphic, but not necessary induced.

#### Definition 6

(Subgraph Isomorphism (SGI)) Given a graph $G=(V_G,E_G)$ and a smaller graph $H=(V_H,E_H)$ with $|E_H|= k$, the SGI problem asks if there is an injective homomorphism from *H* to *G*. An instance of SGI is denoted as $(G=(V_G,E_G), H=(V_H,E_H),k)$.

The parameterized version of SGI has parameter $\kappa = |E_H| = k$. Without loss of generality, we assume

$|V_H| \le 2k$, and every vertex of *H* has degree at most *k*.

For our reduction, we shall work with an alternate but equivalent definition of $k$-Clique that is phrased in terms of SGI.

#### Definition 7

($k$-Clique) Given a positive integer *k* and two graphs $G=(V,E)$ and $H=(W,F)$, where *H* is a complete graph on *k* vertices and parameter *k*, the $k$-Clique problem asks if there is an injective homomorphism from *H* to *G*.

#### Definition 8

($r$-VectorSum) Given a collection ${\mathcal {C}} = \{\vec {\eta _1},\ldots , \vec {\eta _N}\}$ of *d* dimensional vectors over ${\mathbb {F}}_2$, a vector $\vec {b} \in {\mathbb {F}}_2^d$, and an integer *r*, the $r$-VectorSum problem asks if there is $I \subseteq [N], |I|= {r}$ such that $\sum _{{j} \in I} \vec {\eta _{j}}=\vec {b}$, where the sum is over ${\mathbb {F}}_2^d$. An instance of $r$-VectorSum is denoted as $(\mathcal {C},\vec {b},d,r)$.

*f*(*r*)-Compact $r$-VectorSum is defined when $d = f(r) \cdot \textsf {poly} (\log N)$, for some $f(r)\ge r$.

## Dichotomy of PSP

In this section we prove the hardness part of the dichotomy theorem (Theorem 1). First in Sect. 3.1, we identify the gadget and its associated properties that are crucial for the reduction. Then, in Sect. 3.2, using this gadget, we show an FPT-reduction from $k$-Clique to Compact PSP.

### Compatible Intersecting Set System Pair

A set system ${{\mathbb {A}}} = (U_A,\mathcal {S}_A)$ is called an (*M*, *N*)-Intersecting set system (ISS), if it contains *M* sets over *N* elements such that every pair $S,T \in \mathcal {S}_A$ intersects.

#### Definition 9

[Compatible ISS pair] Given two ISS ${{\mathbb {A}}}=(U,\mathcal {S}_A)$ and ${{\mathbb {B}}}=(U,\mathcal {S}_B)$ on a universe *U*, we say that $({{\mathbb {A}}},{{\mathbb {B}}})$ is a compatible ISS pair if there exists an efficiently computable (with respect to size of ${{\mathbb {A}}}$ and size of ${{\mathbb {B}}}$) bijection $f: \mathcal {S}_A \rightarrow \mathcal {S}_B$ such that

- (Complement partition) $\forall S \in \mathcal {S}_A$, *S* and *f*(*S*) forms a partition of *U*, and
- (Complement exchange) $\forall S \in \mathcal {S}_A$, ${{\mathbb {A}}}_S:=(U,(\mathcal {S}_A \setminus \{S\}) \cup \{f(S)\})$ is an ISS.

Note that the complement partition property implies that $f(S) ={\bar{S}}$. Since *f* is as bijection, we have $|\mathcal {S}_A| = |\mathcal {S}_B|$, and $\forall T \in S_B$, the set system ${{\mathbb {B}}}_{T}:=(U,(\mathcal {S}_B {\setminus } \{T\}) \cup \{f^{-1}(T)\})$ is also an ISS. Also, for $(S,T) \in (\mathcal {S}_A,\mathcal {S}_B)$ if $S \cup T = U$, then $T=f(S)$. The following lemma efficiently constructs a compatible (*M*, *N*)-ISS pair, which is a key ingredient in our hardness proof.

#### Lemma 1

For even $N \ge 2$, we can compute a compatible (*M*, *N*)-ISS pair $({{\mathbb {A}}},{{\mathbb {B}}})$ with $M \ge 2^{N/2-1}$ in time polynomial in *M* and *N*. Further, ${{\mathbb {B}}} = \overline{{{\mathbb {A}}}}$.

#### Proof

Given even $N \ge 2$, we construct two set systems ${{\mathbb {A}}} =(U,\mathcal {S}_A)$ and ${{\mathbb {B}}}=(U,\mathcal {S}_B)$ greedily as follows. First set $U = [N]$, and unmark all the subsets ${S} \subseteq U$ of size $\nicefrac {N}{2}$. Then, for every subset ${S} \subseteq U, |S|=\nicefrac {N}{2}$ that is unmarked, add *S* to $\mathcal {S}_A$ and ${\bar{S}}$ to $\mathcal {S}_B$, and mark both *S* and ${\bar{S}}$. Note that $|\mathcal {S}_A|= |\mathcal {S}_B|$, and ${{\mathbb {B}}} = \overline{{{\mathbb {A}}}}$. First, we claim that both ${{\mathbb {A}}}$ and ${{\mathbb {B}}}$ are $(|\mathcal {S}_A|, N)$-ISS. Indeed, observe that any $S, T \in \mathcal {S}_A$ $(S,T\in S_B)$ intersects since $|S| = |T| = N/2$ and $T \ne {\bar{S}}$. Next, for lower bounding *M*, we have $|S_A| = |S_B| = \frac{1}{2} {N \atopwithdelims (){N/2}} \ge 2^{N/2-1}$.

The total time to construct $({{\mathbb {A}}},{{\mathbb {B}}})$ is $2^{N} \textsf {poly} (N) = \textsf {poly} (M,N)$. Finally, to see that $({{\mathbb {A}}},{{\mathbb {B}}})$ is a compatible ISS pair, consider the bijection $f: S_A \rightarrow S_B$ such that $f(S) = {\bar{S}}$ for $S \in \mathcal {S}_A$. Then, note that for $S \in \mathcal {S}_A$, ${{\mathbb {A}}}_s = (U,(\mathcal {S}_A {\setminus } \{S\}) \cup \{f(S)\})$ is an ISS. This is because for $S \in \mathcal {S}_A$ and any ${T} \in {\mathcal {S}_A} \setminus \{S\}$, since $|{\bar{S}}| = |{\bar{T}}| = \frac{N}{2}$ and ${\bar{T}} \ne {\bar{S}}$, we have ${\bar{S}} {\setminus } {\bar{T}} \ne \emptyset$. But ${\bar{S}} \cap {T} = {\bar{S}} {\setminus } {\bar{T}} \ne \emptyset$. Hence, *f*(*S*) intersects *T*. $\square$

### W[1]-hardness of *r*-Compact PSP

The hardness result of Theorem 1 follows from the following FPT-reduction from $k$-Clique that yields an *r*-compact instance of PSP and the fact that $k$-Clique is W[1]-hard.

#### Theorem 4

There is an FPT-reduction that, for every instance ${\textbf{I}} = (G=(V,E), H=(W,F),k)$ of $k$-Clique with $|V_G|=n$ and $|E_G|=m$, computes an instance ${\textbf{J}}=({U},\mathcal {S},r)$ of PSP with $|{U}|= \Theta (k^2 \log n)$, $|\mathcal {S}|= n k + m {k \atopwithdelims ()2}$, and $r=k + {k \atopwithdelims ()2}$ such that *G* has a $k$-Clique if and only if ${\textbf{J}}$ has a packing of size *r*.

#### Proof

Let $V = \{v_1,\ldots ,v_n\}$. We fix an ordering of vertices of *V*. For simplicity, we fix the natural ordering, i.e., $v_i < v_j$ if $i < j$. Further, we assume vertices in an edge are ordered in the same way, i.e., $E \ni e=(v_i,v_j)$ if $i < j$.

Similarly, let $$W=\{w_1,\ldots , w_k\}$$.

Let $({{\mathbb {A}}},\overline{{{\mathbb {A}}}})$ be the compatible (*M*, *N*)-ISS pair given by Lemma 1, for $N = 2\lceil \log (n+1) \rceil +2$. We call ${{\mathbb {A}}}= (U_A,\mathcal {S}_A)$ and $\overline{{{\mathbb {A}}}}= (U_A,\textsf{comp}(\mathcal {S}_A))$ as the *base ISS gadgets*. Let $\mathcal {S}_A= \{S_1,\ldots ,S_M\}$. Since $M \ge 2^{N/2-1} > n$, every $v_i \in V$ can be associated with the set $S_i \in \mathcal {S}_A$ corresponding to the index $i \in [M]$. For $v_i \in V$, we call $S_i \in \mathcal {S}_A$ as the *associated* set of $v_i$. We will use $k(k-1)$ different copies of $({{\mathbb {A}}},\overline{{{\mathbb {A}}}})$ to create an instance ${\textbf{J}} = ({U},\mathcal {S},r)$ of PSP as follows. For each ordered pair $(w_j,w_{j'})$ such that $w_j \in W$ and $w_{j'} \in N(w_j)$, we create a copy $({{\mathbb {A}}}^{(j,j')}, \overline{{{\mathbb {A}}}}^{(j,j')})$ of $({{\mathbb {A}}},\overline{{{\mathbb {A}}}})$. Note that we create two distinct copies – $({{\mathbb {A}}}^{(j,j')}, \overline{{{\mathbb {A}}}}^{(j,j')})$ and $({{\mathbb {A}}}^{(j',j)}, \overline{{{\mathbb {A}}}}^{(j',j)})$, one for each ordered pair $(w_j, w_{j'})$ and $(w_{j'}, w_{j})$ respectively.

Further, for each $j \in [k]$, we define a sub universe of $w_j$ as $U^j_A = \bigcup _{w_{j'} \in N(w_j)} U^{(j,j')}_A$. The universe *U* of ${\textbf{J}}$ is the union of all the universes $U^j_A$, i.e.,

$$\begin{aligned} {U}:= \bigcup _{j =1}^{k} U_A^j. \end{aligned}$$

The sets in $\mathcal {S}$ are of two types: *V*-sets and *E*-sets, as defined below.

*V*-*sets:* For $v_i \in V$ and $w_j \in W$, we create a set $S_{v_i\leftrightarrow w_j}$ as follows.

$$\begin{aligned} S_{v_i\leftrightarrow w_j}:= \bigcup _{w_{j'} \in N(w_j)} S_i^{(j,j')}, \end{aligned}$$

where $S_i^{(j,j')}$ is the copy of the associated set of $v_i$ in $({{\mathbb {A}}}^{(j,j')},\overline{{{\mathbb {A}}}}^{(j,j')})$.

*E*-*sets:* For each edge $(v_i,v_{i'}) \in E$ and for each ordered pair $(w_j,w_{j'})$ of *W*,^3^ we create a set $S_{(v_i,v_{i'})\leftrightarrow (w_j,w_{j'})}$ as follows.

$$\begin{aligned} S_{(v_i,v_{i'})\leftrightarrow (w_j,w_{j'})}:= {\bar{S}}_i^{(j,j')} \cup {\bar{S}}_{i'}^{(j',j)}, \end{aligned}$$

where ${\bar{S}}_i^{(j,j')}$ is the complement of the copy of the associated set of $v_i$ in $({{\mathbb {A}}}^{(j,j')},\overline{{{\mathbb {A}}}}^{(j,j')})$, and ${\bar{S}}_{i'}^{(j',j)}$ is the complement of the copy of the associated set of $v_{i'}$ in $({{\mathbb {A}}}^{(j',j)},\overline{{{\mathbb {A}}}}^{(j',j)})$.

*Parameter:* Set $$r:= k + {k \atopwithdelims ()2}$$.

This concludes the construction. Before we prove its correctness, we note the size of the constructed instance, ${\textbf{J}}$. First, $r= \Theta (k^2)$. Then, $|{U}| = \sum _{i=1}^{k} |U^j_{A}| = \sum _{i=1}^{k} (k-1) N = \Theta (k^2\log n),$ and $|\mathcal {S}| = n k + m {k \atopwithdelims ()2}$.

Yes Case (Completeness). Suppose there is a $k$-Clique $Q=\{v_{i_1},\ldots ,v_{i_k}\} \subseteq V$ in *G*, where the vertices of *Q* are in the natural order.

Then, consider the following collection ${\mathcal {T}}$ of *V*-sets and *E*-sets: ${\mathcal {T}}_V:= \bigcup _{j \in [k]} S_{v_{i_j} \leftrightarrow w_j}$ and ${\mathcal {T}}_E:= \bigcup _{(v_{i_j},v_{i_{j'}}) \in E} S_{(v_{i_j},v_{i_{j'}}) \leftrightarrow (w_j,w_{j'})}$, and let ${\mathcal {T}}= {\mathcal {T}}_V \cup {\mathcal {T}}_E$. Note that these sets exist due to our construction and the existence of *Q*. Further, $|{\mathcal {T}}| = |{\mathcal {T}}_V| + |{\mathcal {T}}_E| = k + {k \atopwithdelims ()2}$, as required. Now, we claim that ${\mathcal {T}}$ forms a packing in ${\textbf{J}}$. Towards this goal, note that it is sufficient to show that ${\mathcal {T}}\mid _{U^j_A}$ forms a packing for all $w_j \in W$. Further, it is sufficient to show that ${\mathcal {T}}\mid _{U^{(j,j')}_A}$ forms a packing for $w_j \in W$ and $w_{j'} \in N(w_j)$. However, this is true since

$$\begin{aligned} {\mathcal {T}} \mid _{U^{(j,j')}_A} = {\mathcal {T}}_V \mid _{U^{(j,j')}_A} \cup \; {\mathcal {T}}_E \mid _{U^{(j,j')}_A} = \{S^{(j,j')}_{i_j}, {\bar{S}}^{(j,j')}_{i_j}\}. \end{aligned}$$

No case (Soundness). We prove the contraposition. Suppose there is an *r*-packing ${\mathcal {T}} \subseteq \mathcal {S}$ in ${\textbf{J}}$. Let ${\mathcal {T}}_V \subseteq {\mathcal {T}}$ and ${\mathcal {T}}_E \subseteq {\mathcal {T}}$ be the *V*-sets and *E*-sets of ${\mathcal {T}}$. Note that our construction lets us identify *V*-sets and *E*-sets in *T*. First, observe that $|{\mathcal {T}}_V| = k$ and $|{\mathcal {T}}_E| = {k \atopwithdelims ()2}$. This is because sets in ${\mathcal {T}}_V$ form a packing, and each set in ${\mathcal {T}}_V$ contains $(k-1)N$ elements of $U^j_A$, for some $j \in [k]$, due to the intersecting property. Since $|{U}| = k(k-1)N$, it follows that $|\mathcal {T}_V| \le k$. Similarly, sets in ${\mathcal {T}}_E$ form a packing, and each set in ${\mathcal {T}}_E$ contains 2*N* elements of *U* due to the intersecting property. Hence, $|{\mathcal {T}}_E| \le {k \atopwithdelims ()2}$. Now since $|{\mathcal {T}}_V| + |{\mathcal {T}}_E| = k + {k \atopwithdelims ()2}$, it must be that $|{\mathcal {T}}_V|=k$ and $|{\mathcal {T}}_E| = {k \atopwithdelims ()2}$. The following lemma is crucial for this case. $\square$

#### Lemma 2

(Covering Lemma) ${\mathcal {T}}$ covers *U*.

#### Proof

Note that each set in ${\mathcal {T}}_V$ contains $(k-1)N/2$ elements of *U*, and each set in ${\mathcal {T}}_E$ contains *N* elements of *U*. Hence, ${\mathcal {T}}_V$ contains $k(k-1)N/2$ elements of *U*, and ${\mathcal {T}}_E$ contains ${k \atopwithdelims ()2}N$ elements of *U* because $|{\mathcal {T}}_V| = k$ and $|{\mathcal {T}}_E| = {k \atopwithdelims ()2}$. Since ${\mathcal {T}}$ is a packing, it holds that ${\mathcal {T}}$ covers exactly $k(k-1)N =|{U}|$ elements of *U*. $\square$

Next, we need the following claim that relies on the above covering lemma.

#### Claim 1

For $v_i, v_i' \in V$ and $1 \le j < j'\le k$, $S_{v_i \leftrightarrow w_j},S_{v_{i'} \leftrightarrow w_{j'}} \in {\mathcal {T}}_V$ if and only if $S_{(v_i,v_{i'}) \leftrightarrow (w_j,w_{j'})} \in {\mathcal {T}}_E$.

#### Proof

Suppose $S_{v_i \leftrightarrow w_j},S_{v_{i'} \leftrightarrow w_{j'}} \in {\mathcal {T}}_V$, then note that ${\mathcal {T}}_E \mid _{U_A^{(j,j')}} = {\bar{S}}_i^{(j,j')}$ and ${\mathcal {T}}_E \mid _{U_A^{(j',j)}} = {\bar{S}}_{i'}^{(j',j)}$, since ${\mathcal {T}}$ is a packing covering *U*. Using the covering lemma, it must be that $S_{(v_i,v_\ell ) \leftrightarrow (w_j,w_{j'}) }\in {\mathcal {T}}_E$ for some $v_\ell \ne v_i$. But this means that ${\bar{S}}_{i'}^{(j',j)} = {\mathcal {T}}_E \mid _{U^{(j',j)}_A} = S_{(v_i,v_\ell ) \leftrightarrow (w_j,w_{j'}) } \mid _{U^{(j',j)}_A} = {\bar{S}}_\ell ^{(j',j)}$ as the base gadget is a compatible ISS pair. This implies that $v_\ell = v_{i'}$, as desired. Similarly, suppose $S_{(v_i,v_{i'}) \leftrightarrow (w_j,w_{j'})} \in {\mathcal {T}}_E$, then note that ${\mathcal {T}}_V \mid _{U_A^{(j,j')}} = {\bar{S}}_i^{(j,j')}$ and ${\mathcal {T}}_V \mid _{U_A^{(j',j)}} = {\bar{S}}_{i'}^{(j',j)}$, since ${\mathcal {T}}$ is a packing covering *U*. Hence, using the covering lemma and the fact that the base gadget is a compatible ISS pair, we have that $S_{v_i \leftrightarrow w_j},S_{v_{i'} \leftrightarrow w_{j'}} \in \mathcal {T}_V$. $\square$

A straight-forward application of this claim and the covering lemma helps us prove the following lemma, which asserts that each vertex of *G* has at most one *V*-set in ${\mathcal {T}}_V$, and each edge of *G* has at most one *E*-set in ${\mathcal {T}}_E$.

#### Lemma 3

For each $v_i \in V$, there is at most one *V*-set $S_{v_i\leftrightarrow w_j}$ in ${\mathcal {T}}_V$, for some $w_j \in W$. Similarly, for each edge $(v_i,v_{i'}) \in E$, there is at most one *E*-set $S_{(v_i,v_{i'})\leftrightarrow (w_j,w_{j'})}$ in ${\mathcal {T}}_E$, for $j \ne j' \in [k]$.

#### Proof

Suppose $S_{v_i\leftrightarrow w_j}, S_{v_i\leftrightarrow w_{j'}} \in {\mathcal {T}}_V$, for some $v_i \in V$, and $1 \le j < j' \le k$. Then, using Claim 1, we have that $S_{(v_i,v_{i}) \leftrightarrow (w_j,w_{j'})} \in {\mathcal {T}}_E$, implying $(v_i,v_i) \in E$, a contradiction. Similarly, for some edge $(v_i,v_{i'}) \in E$, suppose $S_{(v_i,v_{i'})\leftrightarrow (w_j,w_{j'})}, S_{(v_i,v_{i'})\leftrightarrow (w_\ell ,w_{\ell '})} \in {\mathcal {T}}_E$, for some $(j,j') \ne (\ell ,\ell ')$. Without loss of generality, assume $j \ne \ell$. Then, since ${\mathcal {T}}_E \mid _{U_A^{(j,j')}} = {\bar{S}}_i^{(j,j')}$ and ${\mathcal {T}}_E \mid _{U_A^{(\ell ,\ell ')}} = {\bar{S}}_{i}^{(\ell ,\ell ')}$, it must be that $S_{v_i \leftrightarrow w_j}, S_{v_i \leftrightarrow w_{\ell }} \in {\mathcal {T}}_V$ due to Claim 1.

This contradicts our previous argument about *V*-sets. $\square$

Now, we finish the proof as follows. Let $V_{\mathcal {T}} \subseteq V$ be the vertices that have their *V*-sets in ${\mathcal {T}}$. Note that $V_{\mathcal {T}}$ is well defined due to the above lemma, and furthermore, $|V_{\mathcal {T}}| = k$. We claim that $V_{\mathcal {T}}$ is isomorphic to $k$-Clique *H*. Consider $v_{i} \ne v_{i'} \in V_{\mathcal {T}}$. Let $S_{v_{i} \leftrightarrow w_j}$ and $S_{v_{i'} \leftrightarrow w_{j'}}$ be the corresponding *V*-sets in ${\mathcal {T}}_V$, for some $w_j \ne w_{j'} \in W$. Then, note that, using Claim 1, it must be that $S_{(v_i,v_{i'}) \leftrightarrow (w_j,w_{j'})} \in {\mathcal {T}}_E$. Hence, we have $(v_i,v_{i'}) \in E$. $\square$

## ETH hardness of *r*-Compact PSP

Towards proving Theorem 2, we will first show the following reduction from SGI to *r*-Compact PSP. The construction is based on the construction of Sect. 3.2 where we showed an FPT reduction from $k$-Clique to *r*-Compact PSP. However, a major issue in extending the construction of Sect. 3.2 to SGI is (proving) Lemma 3, which promised that any *r*-packing contains at most one *V*-set for each vertex in *V*, and at most one *E*-set for each edge in *E*. This is crucial in the soundness proof to recover the isomorphic copy of *G* to *H*.

### Theorem 5

There is a reduction that, for every instance ${\textbf{I}} = (G=(V_G,E_G),H=(V_H,E_H),k)$ of SGI with $|V_G|=n$ and $|E_G|=m$, computes $\mu = O(k!)$ instances ${\textbf{J}}_p=({U}_p,\mathcal {S}_p,r), p \in [\mu ]$ of PSP in time $O(k!)\textsf {poly} (n)$ with $|{U}_p|= \Theta (k \log n)$, $|\mathcal {S}_p|= \Theta (n^2k + mk)$, and $r=\Theta (k)$, such that there is a subgraph of *G* isomorphic to *H* if and only if there is an *r*-packing in at least one of the instances $\{{\textbf{J}}_p\}_{p \in [\mu ]}$.

### Proof

The construction follows the approach of Sect. 3.2 where we showed an FPT reduction from $k$-Clique to *r*-Compact PSP. Let $V_G = \{v_1,\ldots ,v_n\}$. Let $({{\mathbb {A}}},\overline{{{\mathbb {A}}}})$ be the compatible (*M*, *N*)-ISS pair given by Lemma 1, for $N = 2\lceil \log (n+1) \rceil +2$. We call ${{\mathbb {A}}}= (U_A,{\mathcal {S}}_A)$ and $\overline{{{\mathbb {A}}}}= (U_A,\textsf{comp}({\mathcal {S}}_A))$ as the base ISS gadgets. Furthermore, assume an arbitrary ordering on ${\mathcal {S}}_A= \{S^1,\ldots ,S^M\}$. Since $M \ge 2^{N/2-1} > n$, every $v_i \in V_G$ can be associated with the set $S^i \in {\mathcal {S}}_A$ corresponding to the index $i \in [M]$. We say that the $S^i \in S_A$ is *associated* with $v_i \in V_G$. For each ordering $p: V_H \rightarrow [\ell ]$, create an instance ${\textbf{J}}_p = ({U}_p, \mathcal {S}_p,r)$ of *r*-Compact PSP as follows. Rename the vertices of $V_H$ as $\{w_1,\ldots ,w_\ell \}$ with $w_j:= w \in V_H$ such that $p(w)=j$. For each $w_j \in V_H$, create a collection $\mathcal {C}_{w_j}$ of $d(w_j)+1$ many different copies of base ISS gadget ${{\mathbb {A}}}$ (i.e., each has its own distinct universe) as: $\mathcal {C}_{w_j}:=\{{{\mathbb {A}}}_{w_j,0}, \{{{\mathbb {A}}}_{w_j,w_{j'}}\}_{w_{j'} \in N(w_j)} \}$, where ${{\mathbb {A}}}_{w_i,0} = (U_{w_j,0},{\mathcal {S}}_{w_j,0}) \text { and } {{\mathbb {A}}}_{w_j,w_{j'}} = (U_{w_j,w_{j'}},{\mathcal {S}}_{w_j,w_{j'}}).$ Let $U_{w_j} = \cup _{w_{j'} \in N(w_j)} U_{w_j,w_{j'}}$. For each $\mathcal {C}_{w_j}$, let $U_{\mathcal {C}_j} = U_{w_j,0} \cup U_{w_j}$. Now, we define the *universe* ${U}_p$ of ${\textbf{J}}_p$ as ${U}_p = \bigcup _{j \in [\ell ]} U_{\mathcal {C}_j}$. $\square$

The sets in $\mathcal {S}_p$ are of two types: *V*-sets and *E*-sets as defined below. For $v_i \in V_G$ and $w_j \in V_H$, let $S^i_{w_j}:= \cup _{w_{j'} \in N(w_j)} {S}^i_{w_j,w_{j'}}$. Recall that for $v_i \in V_G$ and $(w_j,w_{j'}) \in E_H$, the set ${S}^i_{w_j,w_{j'}}$ is the copy of the associated set ${S}^i$ in ${\mathcal {S}}_{w_j,w_{j'}}$ of ISS ${{\mathbb {A}}}_{w_j,w_{j'}}=(U_{w_j,w_{j'}},{\mathcal {S}}_{w_j,w_{j'}})$.

*V*-*sets:* For each $v_i \in V_G$, for each $w_j \in \{w_1,\ldots , w_{\ell -1}\}$, and for each $v_{i'} \in V_G, i' > i$, add a set $S_{v_i \leftrightarrow w_j, v_{i'}}$ to $\mathcal {S}_p$ such that

$$\begin{aligned} S_{v_i \leftrightarrow w_j, v_{i'}}:= {{S}}^{i}_{w_j,0} \; \cup \; S^i_{w_j} \; \cup \; \bar{{S}}^{i'}_{w_{j+1},0} \end{aligned}$$

Furthermore, for each $v_i \in V_G$, and for each $v_{i'} \in V_G, i' < i$, add a set $S_{v_i \leftrightarrow w_\ell , v_{i'}}$ to $\mathcal {S}_p$ such that

$$\begin{aligned} S_{v_i \leftrightarrow w_\ell , v_{i'}}:= {{S}}^{i}_{w_\ell ,0} \; \cup \; S^i_{w_\ell } \; \cup \; \bar{{S}}^{i'}_{w_{1},0} \end{aligned}$$

*E*-*sets:* For each edge $(v_i,v_{i'}) \in E_G$ and each edge $(w_j,w_{j'}) \in E_H$, add a set $S_{(v_i,v_{i'}) \leftrightarrow (w_j,w_{j'})}$ to $\mathcal {S}_p$ such that

$$\begin{aligned} S_{(v_i,v_{i'}) \leftrightarrow (w_j,w_{j'})}:= \bar{{S}}^i_{w_j,w_{j'}} \cup \bar{{S}}^{i'}_{w_{j'},w_j} \end{aligned}$$

*Parameter:* Set $$r:= k + \ell $$.

This concludes the construction. Before we prove its correctness, we note the size of the constructed instance ${\textbf{J}}_p$. First, $r= \Theta (k)$, since $\ell \le 2k$. Then, $|{U}_p| = \sum _{i=1}^{\ell } |U_{\mathcal {C}_i}| = \sum _{i=1}^{\ell } (d(v_i) + 1) N = \Theta (k\log n),$ and $|\mathcal {S}_p| = \Theta ( n^2 \ell + mk) = \Theta (n^2k)$.

### Yes Case (Completeness)

Suppose there is a subgraph $G'=(V_{G'},E_{G'})$ of *G* that is isomorphic to *H* with injection $\phi :V_{H} \rightarrow V_{G'}$. Let $V_{G'}=\{v_{i_1},v_{i_2},\ldots , v_{i_\ell }\} \subseteq [n]$ such that $i_1<i_{2}<\cdots <i_{\ell }$. Relabel the vertices of *H* as $\{w_1,\ldots ,w_\ell \}$, where $w_j:= \phi ^{-1}(v_{i_j}), j \in [\ell ]$. Now, consider the ordering *p* of $V_H$ such that $p(w_j)=j$, for $j \in [\ell ]$, and fix the corresponding instance ${\textbf{J}}_p=({U}_p,\mathcal {S}_p,r)$. Consider the following collection of *V*-sets and *E*-sets: ${\mathcal {T}}_V:= \bigcup _{j \in [\ell ]} S_{v_{i_j} \leftrightarrow w_j, v_{i_{j+1}}}$ and ${\mathcal {T}}_E:= \bigcup _{(w_j,w_{j'}) \in E_H} S_{(v_{i_j}, v_{i_{j'}}) \leftrightarrow (w_j,w_{j'})}.$ Let ${\mathcal {T}}={\mathcal {T}}_V \cup {\mathcal {T}}_E$. Note that for this choice of *p*, we have ${\mathcal {T}}_V \subseteq \mathcal {S}_p$ due to construction, and ${\mathcal {T}}_E \subseteq \mathcal {S}_p$ due to $\phi$, and hence ${\mathcal {T}} \subseteq \mathcal {S}_p$. Further, $|{\mathcal {T}}| = |{\mathcal {T}}_V| + |{\mathcal {T}}_E| = \ell + k=r$, as required. Now, we claim that ${\mathcal {T}}$ forms a packing in ${\textbf{J}}_p$. Towards this goal, note that it is sufficient to show that the sets in ${\mathcal {T}}\mid _{U_{\mathcal {C}_j}}$ are mutually disjoint, for all $j \in [\ell ]$. To this end, it is sufficient to show that both ${\mathcal {T}} \mid _{U_{w_j,0}}$ and ${\mathcal {T}} \mid _{U_{w_j}}$ are packing, for all $j \in [\ell ]$. Fix $j \in [\ell ]$, and consider the following cases:

1. ${\mathcal {T}} \mid _{U_{w_j,0}}:$ Since ${\mathcal {T}}_E \mid _{U_{w_j,0}} = \emptyset$ by construction, we focus on ${\mathcal {T}}_V \mid _{U_{w_j,0}}$. But, ${\mathcal {T}}_V \mid _{U_{w_j,0}}$ is a packing since,
  $$\begin{aligned} {\mathcal {T}}_V \mid _{U_{w_j,0}} = {\left\{ \begin{array}{ll} \{S_{v_{i_{j-1}} \leftrightarrow {w_{j-1}}, v_{i_j}}\mid _{U_{w_j,0}}, S_{{i_j} \leftrightarrow w_j, v_{i_{j+1}}}\mid _{U_{w_j,0}}\} = \{{\bar{S}}^{{i_j}}_{w_j,0}, S^{{i_j}}_{w_j,0}\}, \quad \text {if }i \ne 1 \\ \{S_{v_{i_\ell } \leftrightarrow {w_{\ell }}, v_{i_1}}\mid _{U_{w_1,0}}, S_{{i_1} \leftrightarrow w_1, v_{i_2}}\mid _{U_{w_1,0}}\}=\{{\bar{S}}^{{i_1}}_{w_1,0}, S^{{i_1}}_{w_1,0}\}, \quad \text { if }i = 1. \end{array}\right. } \end{aligned}$$
2. ${\mathcal {T}}\mid _{U_{w_j}}:$ It is sufficient to show that ${\mathcal {T}} \mid _{U_{w_j,w_{j'}}}$ is a packing, $\forall w_{j'} \in N(w_j)$. But this follows since, $\forall w_{j'} \in N(w_j)$,
  $$\begin{aligned} {\mathcal {T}} \mid _{U_{w_j,w_{j'}}}&= {\mathcal {T}}_V \mid _{U_{w_j,w_{j'}}} \cup \; {\mathcal {T}}_E \mid _{U_{w_j,w_{j'}}} \\&= \{S_{v_{i_j}\leftrightarrow w_j, v_{i_{j+1}}}\mid _{U_{w_j,w_{j'}}}, S_{(v_{i_j},v_{i_{j'}}) \leftrightarrow (w_j,w_{j'})}\mid _{U_{w_j,w_{j'}}}\} = \{S^{{i_j}}_{w_j,w_{j'}},{\bar{S}}^{{i_j}}_{w_j,w_{j'}} \}. \end{aligned}$$

### No Case (Soundness)

We prove the contraposition. Suppose there is an *r*-packing ${\mathcal {T}} \subseteq \mathcal {S}_p$ in some instance ${\textbf{J}}_p, p\in [\mu ]$, then we show that there is a subgraph $G_{\mathcal {T}}$ of *G* that is isomorphic to *H*. First note that $p \in [\mu ]$ gives a labeling $\{w_1,\ldots , w_\ell \}$ of $V_H$ such that $w_j=p^{-1}(j)$, for $j\in [\ell ]$. Next, partition ${\mathcal {T}}$ into ${\mathcal {T}}_V$ and ${\mathcal {T}}_E$, such that ${\mathcal {T}}_V$ and ${\mathcal {T}}_E$ correspond to the *V*-sets and *E*-sets of ${\mathcal {T}}$ respectively. This can be easily done since $T \in {\mathcal {T}}$ is a *V*-set if and only if $T\mid _{U_{w_j,0}} = S^{i}_{w_j,0} \in {{\mathbb {A}}}_{w_j,0}$, for some $v_i \in V_G, w_j \in V_H$. Let $U_0 = \{U_{w_j,0}\}_{w_j \in V_H}$ and $U_1 = \{U_{w_j}\}_{w_j \in V_H}$. We claim the following.

#### Lemma 4

$$|{\mathcal {T}}_V| = \ell $$ and $$|{\mathcal {T}}_E| = k$$.

#### Proof

Note that for $T \in {\mathcal {T}}_V$, we have $T\mid _{U_0} = S^{i}_{w_j,0} {\cup \;} {\bar{S}}^{i'}_{w_{j+1},0}$, for some $v_i,v_{i'} \in V_G$ and $w_j, w_{j+1} \in V_H$. Hence, it follows that $|T\mid _{U_0}| = N$. Since $|U_0| = \ell N$ and ${\mathcal {T}}_V$ is a packing, we have $|{\mathcal {T}}_V| \le \ell$. For bounding $|{\mathcal {T}}_E|$, consider $T\in {\mathcal {T}}_E$, and note that $T \mid _{U_1} = {\bar{S}}^i_{w_j,w_{j'}} \cup \; {\bar{S}}^{i'}_{w_{j'},w_j}$, for some $(v_i,v_{i'}) \in E_G$ and $(w_j,w_{j'}) \in E_H$. But also note that ${\bar{S}}^i_{w_j,w_{j'}}$ is a set in the ISS $\overline{{{\mathbb {A}}}}_{w_j,w_{j'}}$ and ${\bar{S}}^{i'}_{w_{j'},w_{j}}$ is a set in the ISS $\overline{{{\mathbb {A}}}}_{w_{j'},w_j}$. Hence, by the virtue of ${\mathcal {T}}_E$ being a packing and using the facts that $U_1$ is the union of universes of 2*k* many base ISS $\{\overline{{{\mathbb {A}}}}_{w_j,w_{j'}}\}_{w_j \in V_H, w_{j'} \in N(v)}$, and each $T \in {\mathcal {T}}_E$ contains sets from two of such ISS, it follows $|{\mathcal {T}}_E| \le k$. Finally, $|{\mathcal {T}}| = r = \ell + k$ implies $|{\mathcal {T}}_V| = \ell$ and $|{\mathcal {T}}_E| = k$. $\square$

Since ${{\mathbb {A}}}_{w_j,0}=(U_{w_j,0}, S_{w_j,0}), j \in [\ell ]$ is an ISS, we can relabel the sets in ${\mathcal {T}}_V$ as ${\mathcal {T}}_V = \{T^1_{V},\ldots , T^\ell _{V}\}$, where $T^j_V:= T \in T_V$ such that $T\mid _{U_0} {=} S^i_{w_j,0}$, for some $S^i_{w_j,0} \in {\mathcal {S}}_{w_j,0}$. The following lemma is our key ingredient.

#### Lemma 5

(Covering Lemma) ${\mathcal {T}}$ covers the whole universe ${U}_p$.

#### Proof

Since ${U}_p = U_0 \cup U_1$, we will show that ${\mathcal {T}}\mid _{U_t}$ covers $U_t$, for $t=\{0,1\}$. For $U_0$, note that ${\mathcal {T}}\mid _{U_0} = {\mathcal {T}}_V\mid _{U_0}$ by construction. For $T^j_V\in {\mathcal {T}}_V$, we have $|T^j_V\mid _{U_0}| = N$ due to complement partition property of $({{\mathbb {A}}}_{w_j,0},\overline{{{\mathbb {A}}}}_{w_j,0})$. Since ${\mathcal {T}}_V$ forms a packing, we have that $|\cup _{j \in [\ell ]} T^j_V \mid _{U_0}| = \ell N = |U_0|$, as desired. Next, we have $|U_1| = 2kN$. Consider $T^j_V\in {\mathcal {T}}_V$ and notice $|T^j_V\mid _{U_1}|=\frac{N}{2}d(w_j)$ since $T^j_V \mid _{U_1} = S^i_{w_j}$, for some $v_i \in V_G$. Since ${\mathcal {T}}_V$ forms a packing, we have $|\bigcup _{j \in [\ell ]} T^j_V \mid _{U_1}|= \sum _{j=1}^{\ell } |T^j_V\mid _{U_1}| = kN$. Now consider $T = S_{(v_i,v_{i'}) \leftrightarrow (w_j,w_{j'})} \in {\mathcal {T}}_E$, for some $(v_i,v_{i'}) \in E_G$ and $(w_j,w_{j'}) \in E_H$. Since $T\mid _{U_1} = {\bar{S}}^i_{w_j,w_{j'}} {\cup \;} {\bar{S}}^{i'}_{w_{j'},w_{j}}$, we have $|T\mid _{U_1}| = N$. As ${\mathcal {T}}_E$ forms a packing, we have $|\bigcup _{T \in {\mathcal {T}}_E} T \mid _{U_1}| = \sum _{T \in {\mathcal {T}}_E} |T\mid _{U_1}| = kN$. Finally, ${\mathcal {T}}$ being a packing, we have $| {\mathcal {T}} \mid _{U_1}| = |\bigcup _{i \in [\ell ]} T^i_V \mid _{U_1}| + |\bigcup _{T \in {\mathcal {T}}_E} T \mid _{U_1}| = 2kN = |U_1|$ as desired.

$$\square $$

We say $v_i \in V_G$ *is mapped by* $\mathcal {T}$ *to* $w_j \in V_H$ if $T^j_V\mid _{U_{w_j,0}} = S^{i}_{w_j,0}$. Let $V_{\mathcal {T}}$ be the set of vertices of *G* mapped by $\mathcal {T}$ to some vertex in *H*. The following lemma asserts that $|V_{\mathcal {T}}|=\ell$.

#### Lemma 6

For each vertex $v_i \in V_G$, there is at most one *V*-set $S_{v_i \leftrightarrow w_j, v_{i'}}$ in ${\mathcal {T}}_V$, for some $w_j \in V_H$ and $v_{i'} \in V_G$.

#### Proof

Consider $v'_j,v'_{j'} \in V_\mathcal {T}$ such that $v'_j$ is mapped by $\mathcal {T}$ to $w_j$ and $v'_{j'}$ is mapped by $\mathcal {T}$ to $w_{j+1}$, for $j \in [\ell -1]$. Then, it is sufficient to show $v'_j < v'_{j'}$. Fix $j \in [\ell -1]$ and consider the universe $U_{w_{j+1},0}$ of ${{\mathbb {A}}}_{w_{j+1},0}$. Then, note that only $T^j_V$ and $T^{j+1}_V$ contain elements of $U_{w_{j+1},0}$. Let $T^j_V = S_{v'_j \leftrightarrow w_j, v'_{j''}}$ for $v'_{j''} > v'_j$, and let $T^{j+1}_V = S_{v'_{j'} \leftrightarrow w_{j+1}, v'_{j'''}}$, for $v'_{j'''} > v'_{j'}$. As ${\mathcal {T}}$ covers $U_{w_{j+1},0}$ (Lemma 5), and using the complement partition property of the compatible ISS pair $({{\mathbb {A}}}_{w_{j+1},0},\overline{{{\mathbb {A}}}}_{w_{j+1},0})$, we have that $v'_{j'} = v'_{j''} > v'_{j'}$. $\square$

#### Lemma 7

For every edge $(v_i,v_{i'}) \in E_G$, there is at most one *E*-set $S_{(v_i,v_{i'}) \leftrightarrow (w_j,w_{j'})}$ in ${\mathcal {T}}_E$, for some $(w_j,w_{j'}) \in E_H$.

#### Proof

Suppose there are two sets $S_{(v_i,v_{i'}) \leftrightarrow (w_j,w_{j'})}, S_{(v_i,v_{i'}) \leftrightarrow (w'_j,w'_{j'})} \in {\mathcal {T}}_E$, for some $(v_i,v_{i'}) \in E_G$. Without loss of generality assume $w_j \ne w'_j$. Then, we will show that $S_{v_i \leftrightarrow w_j, {\hat{w}}}, S_{v_i \leftrightarrow w'_{j'}, {\hat{w}}'} \in {\mathcal {T}}_V$, for some ${\hat{w}},{\hat{w}}' \in V_G$, contradicting Lemma 6. Since $S_{(v_i,v_{i'}) \leftrightarrow (w_j,w_{j'})}, S_{(v_i,v_{i'}) \leftrightarrow (w'_j,w'_{j'})} \in {\mathcal {T}}_E$, it holds that ${\mathcal {T}}_E\mid _{U_{w_j,w_{j'}}} = {\bar{S}}^i_{w_j,w_{j'}}$, and ${\mathcal {T}}_E\mid _{U_{w'_j,w'_{j'}}}={\bar{S}}^i_{w'_j,w'_{j'}}$. As ${\mathcal {T}}$ covers ${U}_p$, in particular, ${\mathcal {T}}$ covers $U_{w_j,w_{j'}}$, it must be that ${\mathcal {T}}_V\mid _{U_{w_j,w_{j'}}} = S^i_{w_j,w_{j'}}$ as $({{\mathbb {A}}}_{w_j,w_{j'}}, \overline{{{\mathbb {A}}}}_{w_j,w_{j'}})$ is a compatible ISS pair. By similar reasoning for $U_{w'_j,w'_{j'}}$, it must be that ${\mathcal {T}}_V\mid _{U_{w'_j,w'_{j'}}} = S^i_{w'_j,w'_{j'}}$. This implies that ${\mathcal {T}}_V \mid _{U_{w_j}} = S^i_{w_j}$ and ${\mathcal {T}}_V \mid _{U_{w'_j}} = S^i_{w'_j}$. Thus, $S_{v_i \leftrightarrow w_j, {\hat{w}}}, S_{v_i \leftrightarrow w'_{j'}, {\hat{w}}'} \in {\mathcal {T}}_V$ for $w_j \ne w'_j$, for some ${\hat{w}},{\hat{w}}' \in V_G$. $\square$

Thus, we can now rename the vertices of $V_\mathcal {T}$ as $V_\mathcal {T}= \{v_{i_1},\ldots , v_{i_\ell }\}$, such that $v_{i_j}$ is mapped by $\mathcal {T}$ to $w_j$. Let $G_{\mathcal {T}} = G[V_{\mathcal {T}}] =(V_{\mathcal {T}},E_{\mathcal {T}})$, be the induced subgraph of *G* on $V_{\mathcal {T}}$. To finish the proof, we claim that $G_{\mathcal {T}}$ is isomorphic to *H* with the injective homomorphism $\phi : V_H \rightarrow V_{{\mathcal {T}}}$ given by $\phi (w_j) = v_{i_j}$, for $j \in [\ell ]$. To this end, we will show that for any $(w_j,w_{j'}) \in E_H$, it holds that $(\phi (w_j),\phi (w_{j'}))= (v_{i_j},v_{i_{j'}}) \in E_{\mathcal {T}}$. Consider the universe $U_{w_j,w_{j'}}$, and note that $T^j_V \mid _{U_{w_j,w_{j'}}}= S^{i_j}_{w_j,w_{j'}}$. As ${\mathcal {T}}$ covers $U_{w_j,w_{j'}}$, it holds that ${\mathcal {T}}_E \mid _{U_{w_j,w_{j'}}} = {\bar{S}}^{i_j}_{w_j,w_{j'}}$ since $({{\mathbb {A}}}_{w_j,w_{j'}}, \overline{{{\mathbb {A}}}}_{w_j,w_{j'}})$ is a compatible ISS pair. Hence $S_{(v_{i_j},v_{i'}) \leftrightarrow (w_j,w_{j'})} \in {\mathcal {T}}_E$, for some $(v_{i_j},v_{i'}) \in E_G$. This implies that ${\mathcal {T}}_E \mid _{U_{w_{j'},w_j}} = {\bar{S}}^{i'}_{v_j,v_i}$. By similar arguments for $U_{w_{j'},w_j}$, we have that $v_{i'} = v_{i_{j'}}$ as $T^{j'}_V \mid _{U_{w_{j'},w_j}}= S^{i_{j'}}_{w_{j'},w_j}$. Hence $(v_{i_j},v_{i_{j'}}) = (\phi (w_j),\phi (w_{j'})) \in E_{\mathcal {T}}$. $\square$

### Proof of Theorem 2

We need the following lower bound on the running time for SGI.

#### Theorem 6

([19]) If SGI can be solved in time $f(k)|V_G|^{o(k/\log k)}$ time, for any function *f*, then ETH fails.

Suppose, for the contradiction to the statement of Theorem 2, there is an algorithm A for *r*-Compact PSP running in time $|\mathcal {S}|^{o(r/\log r)}$. Then, we can construct an algorithm B for SGI as follows. Given an instance ${\textbf{I}}= (G=(V_G,E_G), H=(V_H,E_H),k)$ of SGI, B first computes $\mu =O(k!)$ instances ${\textbf{J}}_p = ({U}_p,\mathcal {S}_p,r), p \in [\mu ]$ of *r*-Compact PSP using Theorem 5. Next, B solves each of *O*(*k*!) instances of *r*-Compact PSP using algorithm A, and says yes if and only if A finds an *r*-packing in at least one of $\mu$ instances ${\textbf{J}}_p$ of *r*-Compact PSP. The correctness of Theorem 5 and algorithm A together imply that A correctly decides the SGI problem on instance ${\textbf{I}}$. Observe that the proof of Theorem 5 also finds the subgraph of *G* isomorphic to *H*, if there exists one. The running time of B is bounded by

$$\begin{aligned}&O(k!) \textsf {poly} (n) + O(k!) |\mathcal {S}_p|^{o(r/\log r)} \\&O(k!) \textsf {poly} (n) + O(k! (n^2k)^{o(r/\log r))} \\&O(k!) n^{o(r/\log r)} \qquad \text {using } k \le n\\&O(k!) n^{o(k/\log k)} \qquad \text {using } r = \Theta (k) \end{aligned}$$

Hence, B solves SGI in time $O(k!) n^{o(k/\log k)}$, which contradicts Theorem 6, assuming ETH is true.

## ETH Hardness of *r*-Compact$r$-VectorSum

In this section, we give a proof sketch of Theorem 3.

We will show the following reduction from SGI to compact instances of $r$-VectorSum. Theorem 3 follows using the arguments of Sect. 4.3.

### Theorem 7

There is a reduction that, for every instance ${\textbf{I}} = (G=(V_G,E_G),H=(V_H,E_H),k)$ of SGI with $|V_G|=n$ and $|E_G|=m$, computes $\mu = O(k!)$ instances ${\textbf{L}}_p=(\mathcal {C}_p,\vec {b},d,r), p \in [\mu ]$, of $r$-VectorSum in time $O(k!)\textsf {poly} (n)$ with the following properties:

$$\circ $$
$$d= \Theta (k \log n)$$
$$\circ $$
$$|\mathcal {C}_p|= \Theta (n^2k + mk)$$
$$\circ $$
$$r=\Theta (k)$$

such that there is a subgraph of *G* isomorphic to *H* if and only if there exists $p \in [\mu ]$ such that there are *r* vectors in the instance ${\textbf{L}}_p$ that sum to $\vec {b}$.

### Proof

(Proof Sketch) The first part of the reduction is, in fact, same as that described in Theorem 5, with a simple observation that any optimal packing in the instance generated by Theorem 5 is also a covering. This holds true in No case due to Lemma 5, and it holds true in Yes case due to the complement exchange property of the compatible ISS-pair gadget used in the construction. We call such solution as *exact cover*. In the second part, we transform this instance of Theorem 5 to an instance of $r$-VectorSum. The following definition is useful for the transformation. $\square$

### Definition 10

(Characteristic vector) Let *U* be a universe of *q* elements. Fix an order on the elements of $U = (e_1,\ldots ,e_q)$. For any set $S \subseteq U$, define the characteristic vector $\vec {\chi }_S \in {\mathbb {F}}_2^q$ of *S* as follows. The $t^{th}$ co-ordinate of $\vec {\chi }_S$,

$$\begin{aligned} \vec {\chi }_S(t):= {\left\{ \begin{array}{ll} 1 &  \text { if } e_t \in S,\\ 0 &  \text { if } e_t \notin S. \end{array}\right. } \end{aligned}$$

For every instance ${\textbf{J}}_p=({U}_p,\mathcal {S}_p,r), p \in [\mu ]$ generated by Theorem 5, we create an instance ${\textbf{L}}_p=(\mathcal {C}_p,\vec {b},d,r)$ of $r$-VectorSum as follows. Rename the vertices of $V_H$ as $\{v_1,\ldots ,v_\ell \}$ such that $v_i:= v \in V_H$ such that $p(v)=i$. Note that this induces an ordering on $V_H$ as $v_1< \cdots < v_\ell$. Thus, for $v_i \in V_H$, we have an ordering on $N(v_i) = \{v'_1,\ldots ,v'_{d(v_i)}\}$ as $v'_1< \cdots < v'_{d(v_i)}$. Hence, for $\lambda \in [d(v_i)]$, we call $v'_\gamma$ as the $\lambda ^{th}$ neighbour of $v_i$ if $v'_\gamma$ is the $\lambda ^{th}$ entry in the ordering of $N(v_i)$. Now, for $v_i \in V_H$, we define $\Gamma _i: N(v_i) \leftrightarrow [d(v_i)]$ as $\Gamma _i(v_j):= \lambda \in [d(v_i)]$, such that $v_j$ is the $\lambda ^{th}$ neighbour of $v_i$. Next, we construct vectors corresponding to the sets in $\mathcal {S}_p$. For every *V*-set $S_{\alpha \leftrightarrow v_i}$, for $v_i \in V_H$ and $\alpha \in V_G$, of $\mathcal {S}_p$, define $|{U}_p| + \ell + 2k$ length vector $\vec {\chi }'_{S_{\alpha \leftrightarrow v_i}}$ as follows.

$$\begin{aligned} \vec {\chi }'_{S_{\alpha \leftrightarrow v_i}}(t):= {\left\{ \begin{array}{ll} \vec {\chi }_{S_{\alpha \leftrightarrow v_i}}(t) &  \text { if } t \in [|{U}_p|],\\ 1 &  \text { if } t = |{U}_p| + i, \\ 0 &  \text { otherwise.} \end{array}\right. } \end{aligned}$$

Similarly, for every *E*-set $S_{(\alpha ,\beta ) \leftrightarrow (v_i,v_j)}$, for $(\alpha ,\beta ) \in E_G$ and $(v_i,v_j) \in E_H$, define $|{U}_p| + \ell + 2k$ length vector $\vec {\chi }'_{S_{(\alpha ,\beta ) \leftrightarrow (v_i,v_j)}}$ as follows.

$$\begin{aligned} \vec {\chi }'_{S_{(\alpha ,\beta ) \leftrightarrow (v_i,v_j)}}(t):= {\left\{ \begin{array}{ll} \vec {\chi }_{S_{(\alpha ,\beta ) \leftrightarrow (v_i,v_j)}}(t) &  \text { if } t \in [|{U}_p|],\\ 1 &  \text { if } t = |{U}_p| + \ell + \sum _{\rho =1}^{i-1} d(v_\rho ) + \Gamma _i(j), \\ 1 &  \text { if } t = |{U}_p| + \ell + \sum _{\rho =1}^{j-1} d(v_\rho ) + \Gamma _j(i), \\ 0 &  \text { otherwise.} \end{array}\right. } \end{aligned}$$

Now consider the instance ${\textbf{L}}_p=(\mathcal {C}_p,\vec {b},d,r)$, where

- $$d:= |{U}_p| + \ell + 2k$$
- $$\mathcal {C}_p:= \{\vec {\chi '}_S\}_{S \in \mathcal {S}_p}$$
- $\vec {b}:= \vec {1}$, the all ones vector.

Before we prove the correctness, we define some notations. The first $\ell$ bits that we appended to $\vec {\chi }_S$ are called *V*-indicator bits, and the next 2*k* bits are called *E*-indicator bits. The universe corresponding to *V*-indicator bits and *E*-indicator bits is denoted as $U_2$ and $U_3$ respectively.

*Yes Case* From Theorem 5, there is $p \in [\mu ]$ such that ${\textbf{J}}_p=({U}_p,\mathcal {S}_p,r)$ has a *r*-packing $\mathcal {S}'_p \subseteq \mathcal {S}_p$ that covers ${U}_p$. Then, consider the corresponding instance ${\textbf{L}}_p=(\mathcal {C}_p,\vec {b},d,r)$ of $r$-VectorSum. Then, note that

$$\begin{aligned} \sum _{S \in \mathcal {S}'_p} \vec {\chi _S} = \vec {1} = \vec {b} \end{aligned}$$

since $\mathcal {S}'_p$ is a packing covering ${U}_p$. Hence $\{\vec {\chi }_S\}_{S \in \mathcal {S}'_p} \subseteq \mathcal {C}_p$ is a solution to ${\textbf{L}}_p$.

*No Case* Let $W \subseteq {{\mathcal {C}}_p}, |W|=r$, be a solution of ${\textbf{L}}_p$, for some $p \in [\mu ]$. Let ${\mathcal {T}} \subseteq \mathcal {S}_p$ be the corresponding collection of sets in ${\textbf{J}}_p$ to *W*. Let ${\mathcal {T}}_V$ and ${\mathcal {T}}_E$ be the *V*-sets and *E*-sets of ${\mathcal {T}}$ respectively. Let $W_V$ and $W_E$ be the set of vectors corresponding to ${\mathcal {T}}_V$ and ${\mathcal {T}}_E$ respectively. We say vectors in $W_V$ and $W_E$ as *V*-vectors and *E*-vectors respectively. Note that $W= W_V {\dot{\cup }} W_E$ as ${\mathcal {T}}={\mathcal {T}}_V {\dot{\cup }} {\mathcal {T}}_E$. The following lemma is equivalent to Lemma 4.

### Lemma 8

$$|W_V| = \ell $$ and $$|W_E| = k$$.

### Proof

First consider $W_V$, and note that only *V*-vectors have *V*-indicator bits set to 1. Since a *V*-vector has at most one *V*-indicator bit set to 1, and there are $\ell$ *V*-indicator bits set to 1 in $\vec {b}$, it follows that $|W_V| \ge \ell$. Now consider $W_E$, and note that only *E*-vectors have *E*-indicator bits set to 1. Since a *E*-vector has at most two *E*-indicator bit set to 1, and there are 2*k* *E*-indicator bits set to 1 in $\vec {b}$, it follows that $|W_E| \ge k$. Since, $|W| = \ell + k$, we have $|W_V| =\ell$ and $|W_E|=k$. $\square$

Now we claim that ${\mathcal {T}}$ is an exact *r*-cover in ${\textbf{J}}_p$. It is sufficient to show ${\mathcal {T}}$ is an *r*-packing since ${\mathcal {T}}$ covers ${U}_p$. To this end, note that Lemma 8 implies that $|{\mathcal {T}}_V|=\ell$ and $|{\mathcal {T}}_E| = k$. This implies that that ${\mathcal {T}}\mid _{U_2}$ and ${\mathcal {T}}\mid _{U_3}$ is a packing. Hence, ${\mathcal {T}}\mid _{U_2 \cup U_3}$ is a packing. Let $U'_0:= U_0 \cup U_2 \cup U_3$ and $U'_1:= U_1 \cup U_2 \cup U_3$. Next consider ${\mathcal {T}}_V$ and note that each set in ${\mathcal {T}}_V$ covers exactly *N* elements of $U_0$. Thus, the total number of elements covered by ${\mathcal {T}}_V$ is at most $\ell N$. But then, since ${\mathcal {T}}_V$ covers $U_0$ and $|U_0| = \ell N$, it follows that the every set in ${\mathcal {T}}_V$ must cover different elements of $U_0$. Hence, $\mathcal {T}_V\mid _{U'_0}$ is a packing. On the other hand, $\mathcal {T}_V$ covers at most *kN* elements of $U_1$ as each set in $\mathcal {T}_V$ covers exactly $\frac{N}{2} d(v_i)$ elements of $U_1$, for some $v_i \in V_H$. Since, ${\mathcal {T}}$ covers $U_1$, it must be that ${\mathcal {T}}_E$ must cover at least *kN* elements of $U_1$, as $|U_1|=2kN$. Since each set in ${\mathcal {T}}_E$ covers *N* elements of $U_1$, from Lemma 8 it follows that ${\mathcal {T}}_E$ covers at most *kN* elements of $U_1$. Thus, the sets in ${\mathcal {T}}_E$ must cover different elements of $U_1$, and hence ${\mathcal {T}}_E\mid _{U'_1}$ is a packing. However, ${\mathcal {T}}_E\mid _{U'_1} = {\mathcal {T}}_E$ since sets in ${\mathcal {T}}_E$ only contain elements of $U'_1$. Hence, ${\mathcal {T}}_E$ is a packing. This means that $\mathcal {T}_V$ must cover at least *kN* elements of $U_1$. Then, it follows that ${\mathcal {T}}_V\mid _{U'_1} \cup {\mathcal {T}}_E\mid _{U'_1} = {\mathcal {T}}\mid _{U'_1}$ is a packing since we observed above that ${\mathcal {T}}_V$ covers at most *kN* elements of $U_1$. Thus, ${\mathcal {T}}$ is a packing as ${\mathcal {T}}\mid _{U_0} = {\mathcal {T}}_V\mid _{U_0} \cup {\mathcal {T}}_E\mid _{U_0} = {\mathcal {T}}_V\mid _{U_0}$ is also a packing.

Now since ${\mathcal {T}}$ is an exact *r*-cover of ${U}_p$, we can use the No case of Theorem 5 to recover the isomorphic subgraph of *G* to *H*, which finishes the proof. $\square$

## Footnotes

1 In fact there is another way to define compactness: when $|\mathcal {S}| = f(r) \cdot \textsf {poly} (\log |{U}|)$. However in this case, the *enumerative algorithm* running in time $O^{*}(|\mathcal {S}|^r)$ is already fixed parameter tractable [15]. Thus, the interesting case is when the universe is compact, which is the case we will be focusing on.

2 also known as homomorphic.

3 Note that we create one *E*-set for $(w_j,w_{j'})$ and one *E*-set for $(w_{j'},w_{j})$.

## References

[1] Chataigner, F., Manić, G., Wakabayashi, Y., Yuster, R.: Approximation algorithms and hardness results for the clique packing problem. Discrete Appl. Math. **157**(7), 1396–1406 (2009)

[2] Hassin, R., Rubinstein, S.: An approximation algorithm for maximum triangle packing. Discrete Appl. Math. **154**(6), 971–979 (2006)

[3] Ausiello, G., D’Atri, A., Protasi, M.: Structure preserving reductions among convex optimization problems. J. Comput. Syst. Sci. **21**(1), 136–153 (1980)

[4] Hastad, J.: Clique is hard to approximate within n1-. In: Proceedings of the 37th Annual Symposium on Foundations of Computer Science. FOCS ’96, p. 627. IEEE Computer Society, USA (1996)

[5] Halldórsson, M.M., Kratochvıl, J., Telle, J.A.: Independent sets with domination constraints. Discrete Appl. Math. **99**(1–3), 39–54 (2000)

[6] Austrin, P., Khot, S., Safra, M.: Inapproximability of vertex cover and independent set in bounded degree graphs. In: 2009 24th Annual IEEE Conference on Computational Complexity, pp. 74–80. IEEE (2009)

[7] Bansal, N., Gupta, A., Guruganesh, G.: On the Lovász theta function for independent sets in sparse graphs. SIAM J. Comput. **47**(3), 1039–1055 (2018)

[8] Hazan, E., Safra, S., Schwartz, O.: On the complexity of approximating k-set packing. Comput. Complex. **15**(1), 20–39 (2006)

[9] Chan, Y.H., Lau, L.C.: On linear and semidefinite programming relaxations for hypergraph matching. Math. Program. **135**(1–2), 123–148 (2012)

[10] Cygan, M.: Improved approximation for 3-dimensional matching via bounded pathwidth local search. In: 2013 IEEE 54th Annual Symposium on Foundations of Computer Science, pp. 509–518. IEEE (2013)

[11] Downey, R.G., Fellows, M.R.: Parameterized Complexity. Springer, New York (2012)

[12] Cygan, M., Fomin, F.V., Kowalik, L., Lokshtanov, D., Marx, D., Pilipczuk, M., Pilipczuk, M., Saurabh, S.: Parameterized Algorithms, 1st edn. Springer, Berlin (2015)

[13] Chalermsook, P., Cygan, M., Kortsarz, G., Laekhanukit, B., Manurangsi, P., Nanongkai, D., Trevisan, L.: From Gap-ETH to FPT-inapproximability: clique, dominating set, and more. In: 2017 IEEE 58th Annual Symposium on Foundations of Computer Science (FOCS), pp. 743–754. IEEE (2017)

[14] S., K.C., Khot, S.: Almost Polynomial Factor Inapproximability for Parameterized k-Clique (2021)

[15] Cai, L., Juedes, D.: Subexponential parameterized algorithms collapse the w-hierarchy. In: International Colloquium on Automata, Languages, and Programming, pp. 273–284. Springer (2001)

[16] Bhattacharyya, A., Gadekar, A., Ghoshal, S., Saket, R.: On the hardness of learning sparse parities. In: Sankowski, P., Zaroliagis, C. (eds.) 24th Annual European Symposium on Algorithms (ESA 2016). Leibniz International Proceedings in Informatics (LIPIcs), vol. 57, pp. 11–11117. Schloss Dagstuhl–Leibniz-Zentrum fuer Informatik, Dagstuhl, Germany (2016). 10.4230/LIPIcs.ESA.2016.11 . http://drops.dagstuhl.de/opus/volltexte/2016/6362

[17] Lin, B.: A simple gap-producing reduction for the parameterized set cover problem. In: Baier, C., Chatzigiannakis, I., Flocchini, P., Leonardi, S. (eds.) 46th International Colloquium on Automata, Languages, and Programming (ICALP 2019). Leibniz International Proceedings in Informatics (LIPIcs), vol. 132, pp. 81–18115. Schloss Dagstuhl–Leibniz-Zentrum fuer Informatik, Dagstuhl, Germany (2019). 10.4230/LIPIcs.ICALP.2019.81 . http://drops.dagstuhl.de/opus/volltexte/2019/10657

[18] Björklund, A., Husfeldt, T., Koivisto, M.: Set partitioning via inclusion-exclusion. SIAM J. Comput. **39**(2), 546–563 (2009). 10.1137/070683933

[19] Marx, D.: Can you beat treewidth? In: 48th Annual IEEE Symposium on Foundations of Computer Science (FOCS’07), pp. 169–179. IEEE (2007)

[20] Chu, H.: A Tight Lower Bound for Compact Set Packing (2023). 10.48550/ARXIV.2302.09220 . https://arxiv.org/abs/2302.09220

[21] Jones, M., Lokshtanov, D., Ramanujan, M.S., Saurabh, S., Suchý, O.: Parameterized complexity of directed steiner tree on sparse graphs. In: Bodlaender, H.L., Italiano, G.F. (eds.) Algorithms-ESA 2013, pp. 671–682. Springer, Berlin (2013)

[22] S., K.C., Laekhanukit, B., Manurangsi, P.: On the parameterized complexity of approximating dominating set. In: Proceedings of the 50th Annual ACM SIGACT Symposium on Theory of Computing. STOC 2018, pp. 1283–1296. Association for Computing Machinery, New York, NY, USA (2018). 10.1145/3188745.3188896

[23] Pătraşcu, M., Williams, R.: On the possibility of faster sat algorithms. In: Proceedings of the Twenty-first Annual ACM-SIAM Symposium on Discrete Algorithms, pp. 1065–1075. SIAM (2010)

[24] Lin, B.: Constant approximating k-clique is w[1]-hard. In: Proceedings of the 53rd Annual ACM SIGACT Symposium on Theory of Computing. STOC 2021, pp. 1749–1756. Association for Computing Machinery, New York, NY, USA (2021). 10.1145/3406325.3451016

[25] Flum, J., Grohe, M.: Parameterized Complexity Theory. Texts in Theoretical Computer Science. An EATCS Series, Springer, Berlin (2006)

